# Comparative modelling by restraint-based conformational sampling

**DOI:** 10.1186/1472-6807-8-7

**Published:** 2008-01-31

**Authors:** Nicholas Furnham, Paul IW de Bakker, Swanand Gore, David F Burke, Tom L Blundell

**Affiliations:** 1 Department of Biochemistry, Sanger Building, University of Cambridge, 80 Tennis Court Road, Cambridge, CB2 1GA, UK; 2 Harvard Medical School-Partners Healthcare Center for Genetics and Genomics, Brigham and Women's Hospital, 77 Avenue Louis Pasteur, Boston, MA 02115, USA; 3 Department of Zoology, University of Cambridge, Downing Street, Cambridge CB2 3EJ, UK

## Abstract

**Background:**

Although comparative modelling is routinely used to produce three-dimensional models of proteins, very few automated approaches are formulated in a way that allows inclusion of restraints derived from experimental data as well as those from the structures of homologues. Furthermore, proteins are usually described as a single conformer, rather than an ensemble that represents the heterogeneity and inaccuracy of experimentally determined protein structures. Here we address these issues by exploring the application of the restraint-based conformational space search engine, RAPPER, which has previously been developed for rebuilding experimentally defined protein structures and for fitting models to electron density derived from X-ray diffraction analyses.

**Results:**

A new application of RAPPER for comparative modelling uses positional restraints and knowledge-based sampling to generate models with accuracies comparable to other leading modelling tools. Knowledge-based predictions are based on geometrical features of the homologous templates and rules concerning main-chain and side-chain conformations. By directly changing the restraints derived from available templates we estimate the accuracy limits of the method in comparative modelling.

**Conclusion:**

The application of RAPPER to comparative modelling provides an effective means of exploring the conformational space available to a target sequence. Enhanced methods for generating positional restraints can greatly improve structure prediction. Generation of an ensemble of solutions that are consistent with both target sequence and knowledge derived from the template structures provides a more appropriate representation of a structural prediction than a single model. By formulating homologous structural information as sets of restraints we can begin to consider how comparative models might be used to inform conformer generation from sparse experimental data.

## Background

The three-dimensional (3D) structures of proteins provide valuable insights into their biochemical activities and biological functions. The most widely used experimental methods for determining 3D structures, X-ray crystallography and nuclear magnetic resonance (NMR), have limitations in both time and tractability. For X-ray crystallography sufficient quantities of purified proteins may be difficult to produce and to crystallize when obtained [1]. For NMR, proteins are often too large or insufficiently soluble to be tractable [2]. Nevertheless, genome sequencing projects create a continuing need to translate sequence information into structure [3].

Where experimental methods are problematic, theoretical models can often provide valuable information about the structure of interest. Methods that use physical and chemical properties of amino acids together with information about small fragments of already solved structures have had success with smaller proteins but are still limited in accuracy and reliability [4]. However, knowledge-based methods, such as comparative modelling, which exploit information about amino acid substitutions that accumulate during divergent evolution and are compatible with preserving folded state and function [5], have been the most successful in producing good quality models. Comparative modelling approaches, which can be broadly classified as fragment-based, for example COMPOSER [6], 3D-JIGSAW [7] and SWISS-MODEL [8], or restraint-based, for example MODELLER [9] continue to improve [10]. The latest approaches, for example TASSER, use a combination of threading and restraint optimisation by sampling conformational restraints using Monte Carlo methods [11]. Other protocols use Monte Carlo searches in a reduced space determined by restraints from multiple templates and fragments generated from a consensus of results from a number of modelling programs [12]. Nevertheless, recent CASP exercises [13,14] have demonstrated little significant improvement and have identified empirical limits for knowledge-based protein structure prediction, even when the problem of incorrect alignment has been eliminated [15].

We have previously applied the restraint based conformational search engine RAPPER [16] to a number of protein modelling problems where partial structural information was available, including *ab initio *loop modelling [17,18], Cα tracing [19] and modelling into electron density from X-ray crystallographic experiments [20,21]. Here we develop the approach for comparative modelling, focusing on sampling ϕ/ψ torsion angles under spatial restraints derived from knowledge of homologous structures. We assess the limitations of the method by comparing the use of spatial restraints derived from the homologous template structures with that using restraints derived from experimentally defined structures of targets. We show that significant improvements in model accuracy can be achieved by incorporating additional restraints from main chain curvature and torsion and as side chain χ angle conservation derived from the structures of homologues. By generating an ensemble of solutions consistent with both the target sequence and template structures we provide a more appropriate representation of the structure. By formulating homologous structural information as sets of restraints we can begin to consider how comparative models might be used to inform conformer generation from sparse experimental data [22].

## Results and Discussion

We explored a number of different modes of modelling using RAPPER. The principal differences between these modes lie in the information used from the templates to derive the restraints. In order to minimise problems arising from inaccuracy of sequence alignment, we used structure-based alignments from the HOMSTRAD database [23]. For each of 10 targets, models were generated for one member of the family using four homologues, constructing fifteen models using all possible combinations of four, three, two and one homologue(s) as templates. For comparison, models were also built using the standard modelling mode in MODELLER. This combinatorial approach allowed RAPPER to be parameterised and the performance assessed against a variety of templates of varying sequence identity. In order to assess the usefulness of different restraints described below, models were generated for a greater number of targets using a more limited subset of templates based on percentage sequence identity of target and template. Again, for comparison purposes, models were also generated using MODELLER (See Tables 1, 2, 3, 4, 5).

**Table 1:** Templates for Each of the Targets Modelled.

	PID			
Family	1	2	3	4

Parv ^§^*	62.0	55.1	58.7	53.8
1pvaa	5cpv	1pal	5pal	1a75a

Ghf22 ^§^	69.9	35.8	38.2	45.5
1hml	1hfx	1lz3	1lmn	1jug

Cyt3 ^§^	86.9	52.3	40.2	29.9
2cdv	2cym	1wad	3cyr	2cy3

MHC ^§^	59.8	7.3	11.0	8.9
1fv1b	1iakb	1iaka	1fnga	1fv1a

Flav ^§^*	48.8	33.9	28.6	22.5
1flv	1ag9a	2fcr	1akr	5nll

Phos ^§^	36.9	40.2	40.7	34.7
1bp2	1vpi	1aokb	1vapa	1ppa

Fabp ^§^	32.1	29.0	30.5	19.1
1ifc	1hmt	1lif	1opba	1mdc

Resp ^§^	32.2	25.0	22.6	23.5
1kgsa	1b00a	1dz3a	1tmy	1qkka

Asp ^§^*	54.8	40.2	26.0	27.6
3app	4ape	4apr	1smra	1mpp

Glob ^§^*	87.6	26.0	21.1	20.6
1ymb	1emy	2hhbb	1spgb	1ecd

Az	59.4	69.8	53.5	61.7
2azzaa	1joi	1rkra	1cuoa	1jzga

Blm	43	37.1	39.5	42.2
4blma	3blm	1btl	1mfo	1bul

Cytc	57.7	53.2	45.8	32.1
1yea	2pcbb	1ccr	1cry	1cxc

Egf	32.4	27	27	21.6
1esl	1dan1	1dan2	1rfnb	1hcgb

Fn3	34.8	25.3	20.9	22.5
1fnf2	1fnf3	1fnf1	1fnf4	1ten

Gluts	31.4	27.6	28.6	28.1
17gsa	1guka	1gtua	1guha	1gula

Gtp	64.7	48.8	29.9	32.3
1kao	1guaa	3p21	1mh1	1ftn

Igvar-h	46	43.9	42.9	42.6
1faihv	8fabhv	2fbjhv	2fb4hv	7fabhv

Igvar-l	73.7	68.7	57.3	45.4
2fb4lv	2rhelv	2mcglv	8fablv	3hflv

Ltn	89.9	88.5	47.8	41.6
2ltn	1len	1loe	1lte	1avba

Phc	54.4	55	37.3	33.8
1phnb	1liab	1b8db	1allb	1alla

Sh3	33.3	33.3	31.6	29.6
1shg	1shfa	2src	1qcfa	1lcka

Tim	70.3	56.6	44.7	68.5
1amk	5tima	1ypia	1ydva	1tcda

Members of the homologous families (given by their Homstrad abbreviation) used to model the target are shown with the target and its PDB code in the first column. The percentage sequence identity and the PDB code of the four templates used to model on is given in the subsequent columns. The four families used to parameterise are indicated by *, while the ten families that were used to explore the effect of PID are indicated by **§**.

**Table 2:** Fifteen Different Combinations of Templates Used in Exploring the Effect of PID.

Combination	Template 1	Template 2	Template 3	Template 4
1	✓			
2		✓		
3			✓	
4				✓
5	✓	✓		
6	✓		✓	
7	✓			✓
8	·	✓	✓	
9			✓	✓
10		✓		✓
11	✓	✓	✓	
12		✓	✓	✓
13	✓		✓	✓
14	✓		✓	✓
15	✓	✓	✓	✓

To explore the effect of the number and diversity of templates on RAPPER building using the Standard mode of conformer generation, fifteen combinations as shown in the table where used. Template 1 was closest by PID to the target with Template 4 the most remote.

**Table 3:** All-Atom RMSD for RAPPER Models for 15 Combinations of Template to Target.

	Family									
Combination	Asp	Glob	Parv	Cyt3	MHC_II_N	Fabp	Flav	Phoslip	Responce_reg	Ghf22

1	2.07 (99)	1.37 (100)	1.34 (100)	1.11 (100)	1.95 (96)	2.22 (100)	1.50 (100)	2.85 (100)	3.28 (100)	2.61 (100)
2	2.71 (100)	2.87 (100)	1.68 (99)	2.25 (99)	4.62 (98)	2.10 (100)	1.58 (99)	2.73 (100)	7.37 (100)	2.42 (100)
3	3.77 (100)	2.88 (100)	1.38 (100)	2.20 (100)	4.20 (98)	2.56 (100)	5.80 (100)	2.57 (100)	4.34 (100)	2.26 (100)
4	3.47 (100)	2.95 (99)	1.40 (100)	3.94 (99)	4.37 (96)	2.60 (100)	6.49 (98)	2.85 (100)	3.82 (99)	2.51 (100)
5	2.47 (99)	1.62 (100)	1.30 (96)	1.70 (100)	2.74 (98)	2.16 (100)	1.55 (100)	2.70 (100)	3.60 (98)	2.51 (100)
6	2.71 (99)	1.68 (98)	1.18 (95)	1.25 (99)	2.18 (98)	2.16 (100)	1.51 (99)	2.64 (100)	8.40 (88)	2.43 (100)
7	3.08 (99)	1.59 (100)	1.52 (100)	1.46 (100)	1.83 (95)	2.13 (98)	1.70 (100)	2.67 (100)	3.46 (100)	2.52 (100)
8	3.36 (100)	2.94 (98)	1.26 (96)	1.94 (98)	4.00 (95)	2.28 (100)	2.09 (100)	2.50 (100)	3.87 (99)	2.31 (100)
9	3.13 (99)	2.68 (100)	1.63 (93)	2.35 (99)	4.56 (98)	2.19 (98)	2.18 (99)	2.83 (100)	3.77 (100)	2.17 (100)
10	3.71 (99)	2.63 (100)	1.40 (100)	2.27 (98)	4.34 (98)	2.30 (98)	3.45 (93)	2.52 (100)	3.90 (100)	2.17 (100)
11	2.30 (99)	1.74 (98)	1.17 (95)	1.75 (99)	1.98 (95)	2.20 (99)	1.55 (99)	2.65 (100)	3.52 (98)	2.41 (100)
12	2.68 (99)	1.68 (100)	1.53 (93)	2.01 (100)	2.88 (98)	2.05 (98)	1.78 (100)	2.64 (100)	3.72 (99)	2.56 (100)
13	2.80 (99)	2.08 (96)	1.33 (93)	1.77 (99)	2.24 (98)	2.17 (98)	1.78 (99)	2.64 (100)	3.46 (100)	2.50 (100)
14	3.08 (99)	2.52 (96)	1.37 (93)	2.16 (100)	4.11 (95)	2.14 (98)	2.22 (100)	2.78 (100)	3.18 (67)	2.20 (100)
15	2.52 (99)	1.99 (100)	1.35 (93)	1.75 (99)	1.98 (95)	2.01 (98)	1.83 (99)	2.98 (100)	3.50 (100)	2.43 (100)

The all-atom RMSD in Ångstrom's for RAPPER models built in the standard mode for ten families. The combinations of template to target are as indicated in Table 3.2. To provide a fair comparison the percentage of residues used in the RMSD calculation is given in parentheses.

**Table 4:** All-Atom RMSD for Models Built Using Different RAPPER Restraint Derivations and Templates and for Models Built by Modeller.

	**A**	**B**	**C**	**D**	**E**	**F**	**G**	**H**
**Family**	**RAPPER CA-trace**	**RAPPER optimal spatial restraints**	**RAPPER closest template**	**RAPPER all templates**	**CHORAL/ANDANTE + RAPPER**	**RAPPER all templates sampling by PID**	**MODELLER closest**	**MODELLER all templates**

**Aldosered**	1.23	1.47	1.54	1.59	1.41	1.45	1.45	1.23
**Asp**	1.74	1.89	1.97	2.05	1.86	1.90	1.92	1.74
**Az**	1.31	1.37	1.48	1.36	1.35	1.25	1.60	1.31
**Blm**	1.54	1.55	2.00	1.67	1.83	1.95	2.00	1.47
**Cyt3**	1.21	1.12	1.08	1.67	1.18	1.15	1.26	1.19
**Cytc**	1.45	1.52	1.48	2.16	1.54	1.46	1.48	1.48
**Egf**	1.07	2.16	1.83	3.43	2.22	2.14	2.11	2.20
**Fabp**	1.37	1.98	2.20	2.05	2.35	2.21	2.14	2.13
**Flav**	1.24	1.49	1.45	1.75	1.44	1.69	1.61	1.63
**Fn3**	1.37	1.60	2.11	1.98	2.14	2.24	2.03	1.97
**Ghf22**	1.32	2.07	2.58	2.38	2.39	2.45	2.67	2.03
**Glob**	1.33	1.42	1.36	1.97	1.38	1.43	1.35	1.54
**Gluts**	1.25	2.88	3.19	3.66	2.92	2.84	3.19	2.89
**Gtp**	1.33	2.31	2.44	2.62	2.38	2.50	2.44	2.30
**Igvar-h**	1.52	1.72	2.01	3.24	2.49	2.54	2.31	2.98
**Igvar-l**	1.44	1.47	1.60	2.04	1.54	1.61	1.50	1.51
**Ltn**	1.53	1.36	1.36	1.63	1.34	1.41	1.27	1.34
**MHC**	1.58	1.77	1.93	1.91	1.76	1.82	2.04	2.12
**Parv**	1.18	1.40	1.23	1.33	1.32	1.27	1.27	1.14
**Phc**	1.14	1.56	1.68	1.59	1.53	1.58	1.99	1.74
**Phos**	1.34	2.95	2.84	2.65	2.67	2.54	2.67	2.37
**Resp**	1.23	2.69	3.14	3.41	3.06	3.35	3.03	3.26
**Sh3**	1.38	2.09	2.22	2.24	2.21	2.18	2.50	2.07
**Tim**	1.40	1.24	1.42	1.63	1.26	1.33	1.41	1.37

**Mean**	1.35	1.80	1.92	2.17	1.90	1.93	1.97	1.88

**Family**	**RAPPER CA-trace**	**RAPPER optimal spatial restraints**	**RAPPER closest template**	**RAPPER all templates**	**CHORAL/ANDANTE + RAPPER**	**RAPPER all templates sampling by PID**	**MODELLER closest**	**MODELLER all templates**

	**A**	**B**	**C**	**D**	**E**	**F**	**G**	**H**

The all-atom RMSD given in Ångstrom's for RAPPER models built using **A**. Cα-trace mode; **B**. Using optimal spatial restraints mode using based on all templates; **C**. stranded restraint derivation using just the closest template by PID; **D**. stranded restraint derivation using all templates; **E**. restraints predicted by CHORAL and ANDANTE using all templates; **F**. using restraints based on the PID of the templates using all templates; **G**. using MODELLER based on just the closest templates and **H**. using MODELLER based on all the templates.

**Table 5:** Statistical Analysis of Modelling Methods.

**Statistical Analysis**								
Relation	A < B	A < C	A < D	A < E	A < F	A < G	A < H	

Score	3.20E-04	6.25E-05	5.53E-06	4.89E-05	7.61E-05	2.27E-05	1.72E-04	
P 0.01	TRUE	TRUE	TRUE	TRUE	TRUE	TRUE	TRUE	
P 0.05	TRUE	TRUE	TRUE	TRUE	TRUE	TRUE	TRUE	

Relation	B < C	B < D	B < E	B < F	B < G	B < H		

Score	4.05E-03	9.63E-05	1.29E-02	1.84E-02	7.69E-04	1.28E-01		
P 0.01	TRUE	TRUE	FALSE	FALSE	TRUE	FALSE		
P 0.05	TRUE	TRUE	FALSE	FALSE	TRUE	FALSE		

Relation	C < D	C < E	C < F		G < H	F < H	C < G	C < H

Score	7.67E-03	4.00E-01	2.58E-01		5.28E-02	2.99E-01	6.43E-02	2.37E-01
P 0.01	TRUE	FALSE	FALSE		FALSE	FALSE	FALSE	FALSE
P 0.05	TRUE	FALSE	FALSE		FALSE	FALSE	FALSE	FALSE

The statistical significance of the differences in all-atom RMSD between the modelling methods as reported in Table 3.4. The differences are assessed using a t-test of a paired sample between two means. The truth logic given is for p values of 0.01 and 0.05.

### Deriving theoretically optimal restraints

As a control, we also modelled the target structure based on the experimental Cα positions using RAPPER (as previously described [19]); this provides an upper bound on the quality of the models obtainable using Cα coordinates alone from the homologous templates. The root-mean-square deviations (RMSDs) of these models from the corresponding experimentally determined structures are similar in magnitude to the experimental variation in solution structures determined by NMR. For example, the solution structure of α-parvalbumin has an all-atom RMSD of 1.02 ± 0.08Å (excluding the five first and last residues) [24]. Models built using the Cα trace mode of RAPPER – guided by Cα atom coordinates derived from experimental structures – have loop regions with up to 1Å RMSD and organised secondary structural elements with up to 0.5–0.6Å RMSD from the parent structure. A significant proportion of the difference may result from different crystal packing in the target structure and that of the homologues used in the modelling. [25]. The remainder probably represents errors introduced by (imperfect) restraints from homologous structures.

**Figure 1:**
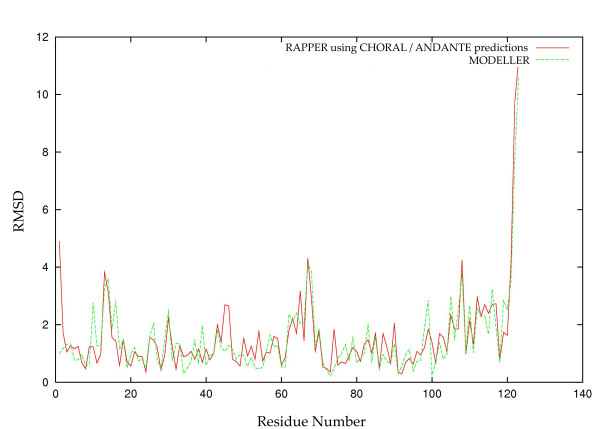
**Contribution to Overall RMSD by Individual Residue**. The per-residue all-atom RMSD for models generated by RAPPER (solid red) and MODELLER (dotted green) for a target of the Ghf22 family. The greatest contribution to the overall RMSD can be seen to be from the C-terminal residues. If these are excluded from the overall RMSD the recalculated RMSD is the same for both modelling procedures.

We calculated the RMSD at each residue in order to identify large local errors which can have an undue influence on the overall RMSD [26]. We calculated other measures such as TM [27], GDT [28] and MaxSub [29] scores as well as the overall RMSD but all failed to identify local regions of inaccuracy in the model. This is illustrated by models for the glycosyl hydrolase family 22 (Ghf22) protein family; Figure 1 shows that the last three residues contribute most to the overall RMSD and this is due to a hook-like conformation of the three C-terminal residues in all available templates, which is not present in the experimental target structure, perhaps due to crystal packing.

Next, we generated the best possible comparative models but now using optimal spatial restraints for each residue. These target structures were superimposed on those of the templates in order to ascertain, for each residue, which template Cα atom is closest to the target. The coordinates of these atoms were then used as the centres for the restraint spheres in an analogous way to the Cα trace mode of RAPPER previously developed for X-ray refinement [19].

Models based on restraints derived from the closest available template (defined by percentage sequence identity) are often close in accuracy to those defined by the Cα-trace model based on the actual structure (all-atom RMSD values shown in Table 4). In many cases, the models show a lower all-atom RMSD than the equivalent models produced by MODELLER, but there are the notable exceptions of Phospholipase A2 (Phos) and the Response Regulator Receiver domain (Resp). In the case of Phospholipase A2 this is due to an insertion of a small section of alpha helical secondary structure flanked by two short loop regions not present in any of the templates. With the lack of any other restraint RAPPER tends to generate expanded loops, while MODELLER's molecular dynamics energy function tends to generate a more compact loop. In the Response Regulator Receiver domain, a section of alpha-helical secondary structure has an incorrect orientation due to a slight extension in one of the flanking loop regions. As with Phospholipase A2 RAPPER minimises the contacts in the flanking loop region which pushes the extension out, resulting in an incorrect orientation of the secondary structural element. For regions which have few short range contacts, RAPPER is provided with few restraints and builds poor models. This might be improved by using secondary structure predictions as restraints to provide a more directed search of the available conformational space.

### Improving on the naïve use of restraints

We explored whether model accuracy can be improved by using multiple templates [30]. We did this by deriving the restraints in three different ways. First, templates were weighted according to their percentage amino acid sequence identity. The size of the restraint sphere derived from each template was varied in size in order to influence the frequency of sampling. This provided a significant improvement in the accuracy (see Tables 4, 5 and 6).

**Table 6:** All-Atom RMSD's for RAPPER Using Two Different Restraint Derivations Compared to Those for MODELLER.

	All-atom RAPPER PDF (Å)	All-atom RAPPER Standard (Å)	Modeller (Å)
Az	1.24	1.62	1.30
Asp	1.89	2.47	1.54
Blm	1.56	2.06	1.55
Cyt3	1.50	1.99	1.19
Cytc	2.07	2.53	2.11
Egf	2.64	2.28	2.64
Fabp	2.18	2.36	2.15
Flav‡	2.30	1.78	1.65
Fn3	2.02	2.35	2.02
Ghf22	1.73	2.50	1.88
Glob‡	2.00	2.10	1.50
Gluts	2.24	3.66	2.84
Gtp	1.88	2.21	1.78
Igvar-h	2.32	2.79	2.36
Igvar-l	1.66	1.78	1.41
Ltn	1.66	1.78	1.41
MHC_II_N	2.61	2.62	2.45
Parv	1.14	1.73	1.21
Phc	1.46	1.81	1.52
Phoslip	1.87	2.56	1.90
Response_reg	2.41	2.64	2.26
Sh3	1.76	2.34	1.88
Tim	1.18	1.58	1.18
Mean	1.87	2.23	1.81

The RMSD for models of each of the family targets generated using RAPPER with restraints derived from PDF's, RAPPER with the standard restraint derivation procedure and MODELLER. The RMSD's for MODLLER and Standard RAPPER differ from the equivalent RMSD's in Tables 3 and 4 as they are calculated over the equivalent number of residues for all models in each family. The two families with difficulties in PDF generation are indicated by ‡.

Secondly, we incorporated information from two newly developed prediction programs as restraints. The first program, CHORAL [31], calculates the curvature and torsion of the main chain residues for each template. Sequences of residues with similar patterns of curvature and torsion are clustered together and scored against the target sequence using environmentally-constrained substitution tables. CHORAL constructs a set of non-overlapping, structurally conserved clusters, which best represent the main chain of the target. Weighting sections of templates by the CHORAL prediction in this way reduces the influence from inappropriate templates on main chain restraints. The second prediction program, ANDANTE [32], predicts the side chain χ angles from likely conservation of those in structures of homologues. These predictions can be used to limit the rotamer search space by RAPPER. The predictions from CHORAL and ANDANTE are presented to RAPPER as possibilities for each template residue defined by ellipsoidal restraints for Cα and side chain centroids. If no prediction is made, then all of the templates are used to generate the restraint ellipsoids. RAPPER models generated using CHORAL/ANDANTE predictions showed significant improvements in the modelling by RAPPER (see Tables 4 and 5).

**Figure 2:**
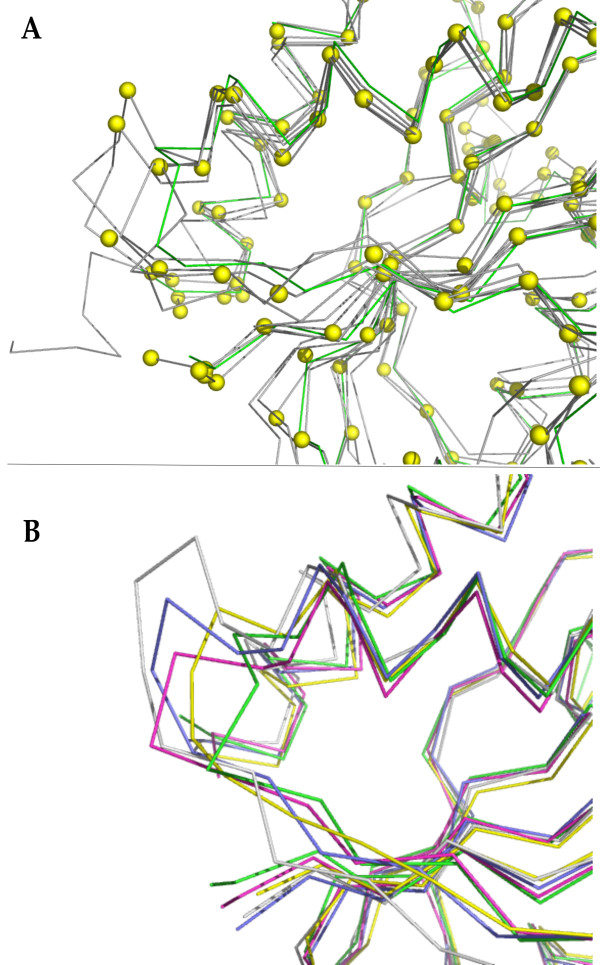
**Problems in Deriving PDF's for Flav Family**. **A**. The superimposed templates in gray with the derived centres of the PDF's shown as yellow spheres. Note the divergent loop on the left. The target structure is shown in green. **B**. The resulting models from different modes of building in RAPPER: RAPPER-PDF in gray, RAPPER-CHORAL in blue, RAPPER-Standard in yellow and MODELLER in pink. The target structure is also shown in green.

Third, we defined restraints from homologues of known structure as 3-D probability density functions, using a local percentage sequence identity calculated over a window of 20 residues. While testing this approach it quickly became obvious that using the standard deviation of the PDF to define the radius of the ellipsoid for the side chain centroid was too restrictive as it prevented effective exploration of a range of rotamer states. Thus the side chain restraint sphere size was set as a default value. A significant improvement in modelling was seen by using restraints generated with a PDF, with P (P = 0.000061 and greater than 0.01) values using a paired means t-test. No overall significant improvement was made compared to MODELLER (P = 0.24 and greater than 0.01). There were a few cases where the PDF-derived restraints led to inaccuracies. For example, when building targets in the flavodoxins (Flav) family, a significant increase in all-atom RMSD comes from the PDF being overly influenced by templates with similar local PID's but significantly different structures (see Figure 2). A similar problem is also observed for the globins (Glob). We had already chosen templates in the relevant functional state, so it was not due to an injudicious choice of templates. For both the flavodoxins and globins arises from differences in conformations, particularly of loops, due to different environments in the crystals.

### Comparing NMR and comparative ensembles

Although NMR methods have led to the generation of ensembles, X-ray and comparative models have usually been presented as single conformers, though often multiple models are generated during the experimental or modelling process. An ensemble of multiple conformers captures more information, as it allows regions to be identified that are structurally variable, representing the intrinsic dynamics of the target structure or uncertainties in the modelling process. In order to examine this, we compared the ensemble generated by RAPPER for 1pvaa as target, using other structures from the α-parvalbumin family (Parv), to the experimentally determined NMR ensemble of the same protein.

**Figure 3:**
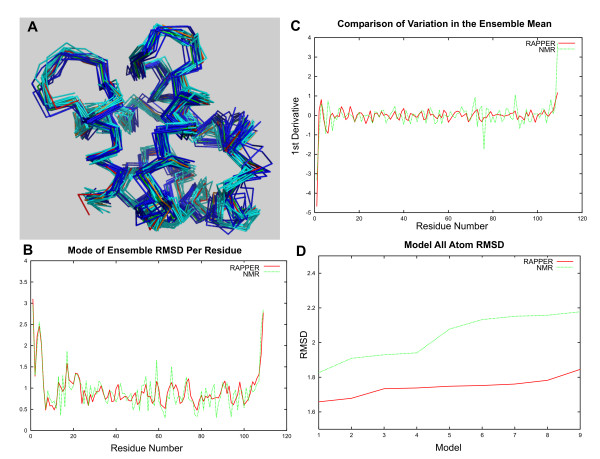
**Comparison of RAPPER and NMR Ensembles to the Crystallographic Model**. Comparison of a RAPPER ensemble of comparative models for the target 1PVA chain A from the Parvalbulmin family with an NMR ensemble, the crystal structure and the deposited representative NMR structure. A: The backbone trace of 9 models from the RAPPER ensemble (cyan) generated by comparative modelling on all targets and the equivalent models generated by NMR (blue). Also shown are the deposited crystal structure (red) and the representative NMR single model (orange). All models are superimposed with reference to the crystal structure. B: The plot of ensemble mean and mode for each residue in the RAPPER ensemble. C: The 1st derivative of the per residue ensemble mean for RAPPER (red) and the NMR ensemble (green). D: The all atom per residue RMSD for the RAPPER representative single model (red) compared to the equivalent single NMR representative model (green).

The RAPPER and NMR ensembles, superimposed on the crystallographic model, are shown in Figure 3A. It can be seen that the two ensembles have similar features with respect to compactness and diversity in different regions of the polypeptide chain, with the comparative modelling ensemble closer to the crystal model than the NMR ensemble. In order to gain more insight into this observation, the mode of the distribution of RMSD for the two ensembles was calculated for each residue. The two curves (Figure 3B) are very similar as shown by a correlation coefficient of 0.66 when comparing the first derivative for each curve (Figure 3C). The fact that the RAPPER ensemble is more similar to the crystallographic model than the NMR ensemble can be seen when the all-atom RMSD is calculated for each of the models in the ensemble (Figure 3D). If the all-atom RMSDs are calculated for the two ensemble representative models, the RAPPER representative model is closer to the crystal structure than the equivalent representative model from the NMR ensemble. The representative model is, in the case of RAPPER, the geometric average of the ensemble, while in the case of NMR it is that chosen by the NMR spectroscopist on deposition to the PDB. Furthermore the RAPPER representative model is always much closer to the crystal structure than any of the individual models that make up the ensemble. The wider variability seen in the NMR ensemble may be due to compaction by crystal packing. Also the crystallographic model is a single time and space averaged representation of the protein in question. This representation may be inadequate in fully explaining the experimental data, especially at medium and lower resolutions [33].

## Conclusion

The differences between the comparative modelling protocol of RAPPER, the Cα-trace models, and most importantly modes that use an optimal restraint network based on knowledge of the target structure demonstrate that there is a limit to which we could hope to build a reliable model based solely on homologous templates using RAPPER. Nevertheless, the restraint networks based on differential geometry, pattern recognition and χ angle conservation described here are all shown to be useful approaches to introducing further structural information.

The application of RAPPER to comparative modelling provides an effective means of exploring the conformational space available to a target sequence. The use of different methods for defining restraints from homologous templates shows that better methods for generating positional restraints can greatly improve structure prediction. Generation of an ensemble of solutions that are consistent with both target sequence and knowledge derived from the template structures provides a more appropriate representation of a structural prediction than a single model.

As we have already demonstrated in generating conformers using low resolution X-ray data[21], RAPPER allows the testing of weak hypotheses and speculations about structures where the ratio of observations to parameters is low. For comparative modelling, where restraints derived from distant homologues or regions of divergent structure are often inaccurate, we have now shown that RAPPER can explore conformational space defined by restraints from varying combinations of templates or secondary structure predictions. This suggests that there might be advantage in integrating restraints derived from knowledge of homologous structures with restraints provided by sparse or low resolution experimental data. Thus information from structures of homologues could be of particular use in generating conformers consistent with low resolution X-ray electron density and electron microscopy density, NMR where there are insufficient observations and small angle X-ray scattering (SAXS). We are now investigating such applications, not only with RAPPER but also with RAPPER-TK [34], which can be used to model not only proteins but also other macromolecules and their complexes.

## Methods

### Modelling data set

In order to develop and test the approach twenty four families were chosen from the HOMSTRAD database [23], representing each of the four main SCOP classes (all α, all β, α + β and α/β). For each family five members were chosen based on maximizing the range of the relative percentage identity (PID) based on sequence (calculated by Malform [35]) and ensuring all the solved structures were of relatively high resolution (greater than 2Å). One member was designated as the target, with the rest acting as the templates. This allowed fifteen combinations of the templates exploiting one to four homologues as targets, so reflecting information from homologues across the range of PID. The data set was sub-divided into three. The first consisted of four families that were used to define the default parameters. The restraint defaults for main chain and side chain restraint sphere size were chosen by iteratively reducing the radii in a combinatorial manner until RAPPER was unable to generate a model. The second set comprising a further six families was used to generate all 15 combinations of template to target. The third set comprises all of the chosen families and were used to test alternative approaches to defining restraints. Table 1 shows the families and their constituent members. The possible combinations of target to templates are given in Table 2. Each of the combinations, including the target, were structurally aligned using COMPARER [36] and annotated by JOY [37]. The resulting alignments were manually corrected, resulting in the best possible alignment and thus minimising any error from an incorrect alignment.

### Modelling procedure for RAPPER

The application of the conformational search engine RAPPER to comparative modelling by satisfaction of spatial restraints was achieved by extending the restraint engine as described for solving the Cα trace problem [19]. From the given alignment a structural superimposition of equivalent residues is made and optimised. A common core was defined from the set of aligned protein structures as the subset of equivalent residue atoms with relatively little structural variation as defined by the Altman-Gerstein algorithm [38] and implemented in RAPPER. Based on this superimposition and alignment, spatial restraints can then be described for each residue of the target sequence. There are four types of spatial restraint:

1 – As RAPPER builds from the N to C termini a bootstrap restraint is required to allow modelling to commence. The bootstrap is defined as the mean position of the Cβ coordinates from the templates, which is made the centre of a restraint sphere, the size of which is user-defined. In building the first two residues a position of the first residue Cβ is taken at a random offset from the mean Cβ coordinate position of the equivalent Cβ of the templates. From this the remaining backbone atom positions can be calculated from the ideal Engh and Huber [39] bond angles and lengths implicit in the RAPPER protein model. A ψ angle is then randomly picked from high-grained residue specific ϕ/ψ propensity tables as well as a random angle for the vector between the first and the second Cβ position. Thus the first peptide bond is generated.

2 – A set of spatial restraints is defined for the backbone (main chain) atoms, principally the Cα atoms. Each is defined as an ellipsoid generated from the union of the set of restraint spheres centred on the equivalent atom position from each of the templates, as defined in equation 1. The size of these spheres is user defined.

$$\|\vec{p}-\vec{O}\|\leq r$$

where $\vec{p}$ is the position of the Cα atom, $\vec{O}$ is the centre of the restraint sphere with radius r.

3 – A similar set of spherical restraints can be defined for the side chain atoms, except that, rather than taking each atom separately, a virtual centroid (as defined in equation 2) of the side chain is calculated and this position is used to centre the restraint sphere. In fact two virtual centroid positions are calculated: a short virtual centroid position which essentially takes into account the atoms up to and including the Cγ position and a long virtual centroid position which accounts for the rest of the side chain.

$$\|\left(\sum_{i}^{N_{sc}}{\vec{p}}_{i}\right)/N_{sc}-\vec{O}\|\leq r$$

where *N*_*sc *_is the number of side chain atoms

4 – A set of spatial restraints is derived for secondary structure elements. Residues are defined to be in elements of secondary structure from consideration of the consensus across the template structures or from secondary structure prediction. The restraints are a combination of restricted ϕ/ψ sampling of the residue specific ϕ/ψ propensity tables to the alpha helical or beta sheet regions of ϕ/ψ space and short range hydrogen bonding distance restraints. Only short range hydrogen bonding is enforced and this primarily in alpha helical regions, although we have now developed algorithms for including more long range restraints (A Karmali and N Furnham, unpublished data).

As well as the specific restraints from homologues, a number of other restraints are also enforced including clash restraints against the framework structure as it is built and distance restraints from ideal bond angles, bond lengths and omega torsion angles. All of the restraints can be propagated along the chain for a user defined distance.

**Figure 4:**
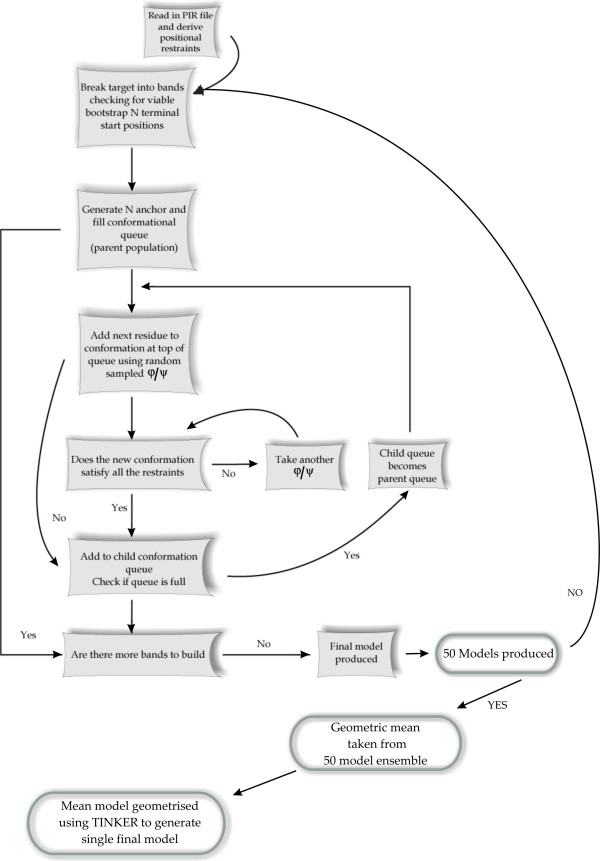
Schematic of RAPPER Conformer Generation Applied to Comparative Modelling.

The standard building process in RAPPER as described previously is used [18,19]. Briefly, the algorithm employs a branch and bound protocol to extend iteratively the polypeptide chain in the N to C-terminal direction. A population of 100 fragments that make up the growing polypeptide chain is maintained, with a maximum of 100,000 attempts to find the 100 solutions to the restraint network at each residue position. As some residues are in rare ϕ/ψ conformations this may still be insufficient to sample effectively the ϕ/ψ space. Thus, to optimise the time spent searching the target sequence is split into a number of fragments, avoiding regions where there is no template information available, but otherwise randomly. A population of 50 models is produced for each target. The geometric average of the model population is calculated in RAPPER. The resultant single model is then re-geometrised by TINKER [40]. The protocol is summarised in Figure 4.

Models were constructed using this standard comparative modelling mode. In each round of building 2Å spheres where enforced for the bootstrap, Cα main chain and side chain restraints. These values were determined from the subset of four families used to parameterise the modelling procedure. This parameterisation was achieved by iterative rounds of building adjusting each of the parameters in a combinatorial approach, starting from a large value and gradually decreasing in 0.5Å increments till the restraints were too strict for a model to be built. The last round where the model could be successfully generated was taken as the optimal parameters.

### RAPPER sampling by PID

The results of modelling using all the templates demonstrate that the approach would benefit from restricting the available search area. This can be simply achieved by weighting towards the restraints derived from the template with the highest PID to the target, which is accomplished by reducing, based on the PID of the template to target, the relative size of the restraint spheres. The range of PID across the available templates is calculated and is divided into four equal sub-ranges. If the PID of the template lies in the top quartile then the user defined restraint sphere radius is enforced. If the PID of the template lies in one of the other three quartiles, then the restraint sphere is reduced by a corresponding factor, with the restraint spheres generated from the template whose PID lies in the lowest quartile being reduced by 60%. In addition the sampling frequency of the restraint sphere generated from the template with the highest PID is enhanced.

### RAPPER using probability density function derived restraints

**Figure 5:**
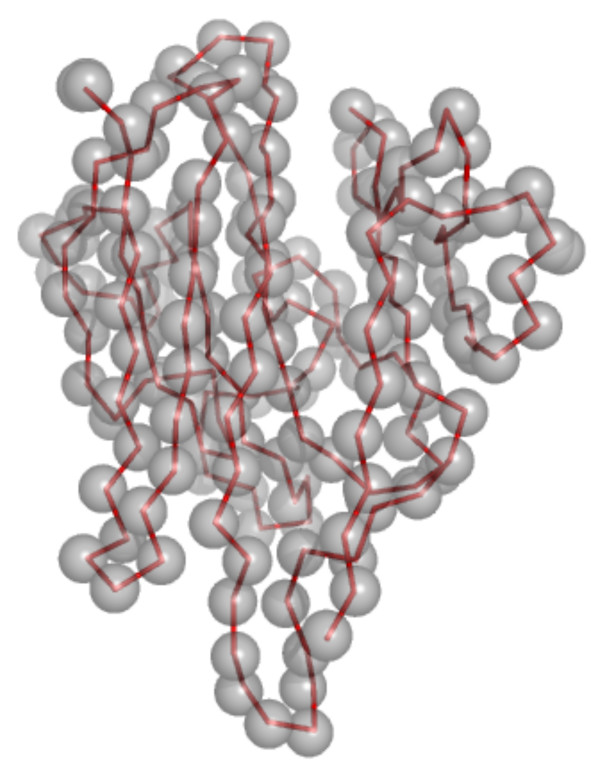
**Centres of PDF's Compared to the Target**. PDF's and target for the Ltn family. The centres of each PDF shown as a space filled sphere with the ribbon trace of the target in red. Note that the size of the sphere does not represent the size of the PDF sphere enforced in RAPPER.

More distantly related homologous structures can be exploited if restraints are formulated as probability density functions (PDF). The position of each atom (or centroid for side chains) can be used to centre a probability function described as a Gaussian distribution, the mean of which is the atom position and the variance is the local PID taken over a window of 20 residues as a

$$PDF_{i}=\frac{Ae^{-\frac{{\left(x-x_{1}\right)}^{2}}{2\sigma_{1}^{2}}}}{\sqrt{2\pi }}$$

where *i *is the position in the template sequence, x_1 _is Cα position of the template and σ_1_^2 ^is inversely proportional to the PID of the template. The sum of the distributions of each of the homologous atom positions is calculated and normalised to generate a PDF (equation 4).

$$P(x)=\int_{-\infty }^{\infty }PDF_{i}(t1)+PDF_{i}(t2)+PDF_{i}(t3)+...$$

where x is the coordinate in question and t is the template. This is done for each of x, y and z coordinates. The resulting mean position of the combined PDF is taken as the centre of the restraint sphere, the radius of which can either be user defined or defined by the standard deviation of the new distribution for each coordinate, which can then be used to define an ellipsoid (see Figure 5).

### RAPPER using CHORAL/ANDANTE predictions

An alternative approach to defining restraints based upon information from homologous structures can be achieved by taking advantage of the predictions of two programs: CHORAL [31] and ANDANTE [32]. CHORAL, an amalgam of differential geometry and pattern recognition algorithms, identifies the clusters of conformers from homologous templates with conserved curvature and torsion that are most likely to represent the core backbone of the target structure. ANDANTE uses environmental-specific substitution probabilities to predict where χ1, χ1 plus χ2, or χ1 plus χ2 plus χ3 can be directly used from a single template to limit the rotamer search space. Thus, RAPPER uses the equivalent template residue(s) predicted to contribute either to the target's core backbone or side chain conformations to generate the restraint network. For example, if CHORAL predicts that residue *i *in the target sequence will have similar backbone conformations to the equivalent residues of template 1 and template 2, the Cα atoms of these two templates are used as the centres of the main chain restraint spheres. Similarly, where ANDANTE predicts that the χ1 plus χ2 of template 2 is most likely to be conserved in the target, the short virtual centroid position is used as the centre of the short side chain restraint sphere. RAPPER then builds through this restraint network in the same manner as the standard method for restraint derivation.

For each target the protocol in the standard comparative modelling procedure is used to produce an ensemble of 50 models; the arithmetic mean is taken and the structure re-geometrised using TINKER [40]. The approach of using CHORAL/ANDANTE predictions allowed tighter restraints of 1Å radius to be universally enforced for both main chains and side chains. Where CHORAL or ANDANTE did not predict conformations for a residue i.e. a variable loop region or where there was no prediction of side chain rotamer, all of the templates were used to generate the restraint network with the larger 2Å radius. The restraint sphere radius in the interface between the conserved core and non-conserved region for the backbone was "funnelled" at the end of the conserved core region (gradually increasing from 1Å to 2Å) and the beginning of the next conserved core region (gradually decreasing from 2Å to 1Å). This provided continuity in the main chain restraint network, ensuring no unrealistic distances were required to be satisfied.

### Baseline Modelling

In addition to the basic comparative mode of RAPPER, further models were constructed in order to estimate the limitations of the method. For example we used the Cα trace mode of RAPPER [19] to rebuild the target based on experimentally observed co-ordinates. We also exploited restraints from secondary structure information, using the actual atomic positions of the Cα atoms of the experimentally resolved target to define the restraint network. Alternatively the template with the minimum distance from its Cα to that of the target was used while ensuring that this was consistent with the previous restraint sphere centre by approximately a Cα-Cα bonds length to define restraints.

### Other modelling programs

The targets were also built using the well established comparative modelling program: MODELLER [41]. Ten models were produced by MODELLER using the standard model-building routine. A single model was automatically selected based on the average between the minimal energy as calculated by MODELLER and minimal steric violations.

## Authors' contributions

NF conducted the data acquisition processing and developed (with PIWDB) the comparative modelling mode of RAPPER. SG provided the PDF code and programming support. DFB participated in study design and aided in the analysis. TLB conceived of the study, participated in its design and coordination and helped to draft the manuscript. All authors read and approved the final manuscript.
